# A Socially Adaptable Framework for Human-Robot Interaction

**DOI:** 10.3389/frobt.2020.00121

**Published:** 2020-10-19

**Authors:** Ana Tanevska, Francesco Rea, Giulio Sandini, Lola Cañamero, Alessandra Sciutti

**Affiliations:** ^1^Department of Robotics, Brain and Cognitive Science, Italian Institute of Technology (IIT), Genova, Italy; ^2^EECAiA Lab, School of Computer Science, University of Hertfordshire, Hatfield, United Kingdom; ^3^Cognitive Architecture for Collaborative Technologies Unit, Italian Institute of Technology (IIT), Genova, Italy; ^4^Neurocybernetics Team, ETIS Lab, CY Cergy Paris Université, ENSEA, CNRS UMR8051, Cergy-Pontoise, France

**Keywords:** human-robot interaction, social adaptability, affective interaction, personalized HRI, emotion recognition

## Abstract

In our everyday lives we regularly engage in complex, personalized, and adaptive interactions with our peers. To recreate the same kind of rich, human-like interactions, a social robot should be aware of our needs and affective states and continuously adapt its behavior to them. Our proposed solution is to have the robot learn how to select the behaviors that would maximize the pleasantness of the interaction for its peers. To make the robot autonomous in its decision making, this process could be guided by an internal motivation system. We wish to investigate how an adaptive robotic framework of this kind would function and personalize to different users. We also wish to explore whether the adaptability and personalization would bring any additional richness to the human-robot interaction (HRI), or whether it would instead bring uncertainty and unpredictability that would not be accepted by the robot's human peers. To this end, we designed a socially adaptive framework for the humanoid robot iCub. As a result, the robot perceives and reuses the affective and interactive signals from the person as input for the adaptation based on internal social motivation. We strive to investigate the value of the generated adaptation in our framework in the context of HRI. In particular, we compare how users will experience interaction with an adaptive versus a non-adaptive social robot. To address these questions, we propose a comparative interaction study with iCub whereby users act as the robot's caretaker, and iCub's social adaptation is guided by an internal comfort level that varies with the stimuli that iCub receives from its caretaker. We investigate and compare how iCub's internal dynamics would be perceived by people, both in a condition when iCub does not personalize its behavior to the person, and in a condition where it is instead adaptive. Finally, we establish the potential benefits that an adaptive framework could bring to the context of repeated interactions with a humanoid robot.

## 1. Introduction

People have a natural predisposition to interact in an adaptive manner with others, by instinctively changing their actions, tones, and speech according to the perceived needs of their peers (Lindblom, 1990; Savidis and Stephanidis, 2009). Moreover, we are not only capable of registering the affective and cognitive state of our partners, but over a prolonged period of interaction we also learn which behaviors are the most appropriate and well-suited for each one individually (Mehrabian and Epstein, 1972). This universal trait that we share regardless of our different personalities is referred to as social adaptation (adaptability) (Terziev and Nichev, 2017). Humans are very often capable of adapting to others, though our personalities may influence the speed and efficacy of the adaptation. This means that in our everyday lives we are accustomed to partake in complex and personalized interactions with our peers.

Translating the ability to personalize interactions into HRI is highly desirable since it would provide user-personalized interaction, a crucial element in many HRI scenarios—interactions with older adults (Kidd et al., 2006; Broadbent et al., 2011; Sharkey, 2014), assistive or rehabilitative robotics (Plaisant et al., 2000; Admoni and Scassellati, 2014; Wood et al., 2017), child-robot interaction (Tanaka and Matsuzoe, 2012; Paiva et al., 2014), collaborative learning (Jimenez et al., 2015; Ramachandran et al., 2016), and many others. For a social robot to be able to recreate this same kind of rich, human-like interaction, it should be aware of our needs and affective states and be capable of continuously adapting its behavior to them (Breazeal and Scassellati, 1999; Cañamero et al., 2006; Kishi et al., 2014; Vaufreydaz et al., 2016; Ahmad et al., 2019).

However, equipping a robot with these functionalities is not a straightforward task. One potentially robust approach for solving this complexity might consist of implementing a framework for the robot supporting social awareness and adaptation (Cangelosi et al., 2010). In other words, the robot would need to be equipped with the basic cognitive functionalities, which would allow it to learn how to select the behaviors maximizing the pleasantness of the interaction for its peers, while being guided by an internal motivation system that would provide autonomy in its decision-making process.

In this direction, the goal of our research was threefold: to attempt to design a cognitive architecture supporting social HRI and implement it on a robotic platform; to study how an adaptive framework of this kind would function when tested in HRI studies with users; and to explore how including the element of adaptability and personalization in a cognitive framework would in reality affect the users. For instance, would it bring an additional richness to the human-robot interaction as hypothesized, or would it only add uncertainty and unpredictability that would not be accepted by the robot's human peers?

In our past works, we have explored adaptation in child-robot interaction (CRI) in the context of switching between different game-based behaviors (Tanevska, 2016). The architecture was affect-based (Tanevska et al., 2018b), and the robot could express three basic emotions (a “happy,” a “sad,” and a “neutral” state) in a simple way. These emotions were affected by the level of engagement the child felt toward the current robot's behavior. The robot aimed to keep the child entertained for longer by learning how the child reacted to the switch between different game modalities. We have since expanded on the core concept of a robot's internal state guiding the adaptation, and have advanced from the discrete emotional states and one-dimensional adaptation to a more robust framework. Starting from the work of Hiolle et al. (2012, 2014) on affective adaptability, we have modified our architecture to utilize as motivation the level of comfort of the robot, which is increasing when the robot is interacting with a person, and decreasing when it is left on its own.

The robotic platform selected for our study was the humanoid robot iCub (Metta et al., 2008), and the scenario for testing the framework's functionalities was inspired by a typical interaction between a toddler and its caregiver, where the toddlers tend to seek the attention of their caretakers after being alone for a while, but as soon as their social need has been saturated they lose interest and turn their attention to something else (Feldman, 2003). The robot therefore acted as a young child, asking the caretaker's company or playing on its own. The human partners could establish and maintain the interaction by touching the robot, showing their face and smiling, or showing toys to the robot. This scenario was deemed suitable for studying some fundamental aspects of interaction (such as initiation and withdrawal) with a fully autonomous robot behavior and very limited constraints to the human activities, as well as in a seemingly naturalistic context. Furthermore, we verified these assumptions over the course of several validation and pilot studies (Tanevska et al., 2019).

In this paper we cover the work we did on developing a cognitive framework for human-robot interaction; we analyze the various challenges encountered during the implementing of the cognitive functionalities and porting the framework on a robotic platform; and finally we present the user studies performed with the iCub robot, focused on understanding how a cognitive framework behaves in a free-form HRI context and whether humans can be aware and appreciate the adaptivity of the robot. The rest of the paper is organized as follows: section 2 gives an overview on the state of art in cognitive architectures, section 3 presents the adaptive framework for our architecture, followed by section 4 which presents the experimental methods applied in our study with iCub. Finally, in sections 5, 6 we present the findings from our study and we touch on our plans for future work.

## 2. Cognitive Architectures

A cognitive agent (be it a natural or an artificial one) should be capable of *autonomously* predicting the future, by relying on *memories* of the past, *perceptions* of the present, and *anticipation* of both the behavior of the world around it as well as of its own actions (Vernon, 2014). Additionally, the cognitive agent needs to allow for the uncertainty of its predictions and *learn* by observing what actually happens after an action, and then assimilating that perceptive input into its knowledge about the world, *adapting* its behavior and manner of doing things in the process.

Following this, cognition can be defined as the process by which an autonomous agent perceives its environment, learns from experience, anticipates the outcome of events, acts to pursue goals, and adapts to changing circumstances.

In the past decades several different cognitive architectures have been proposed and tested on artificial agents in a variety of cognitive tasks—SOAR (Laird, 2012), ACT-R (Anderson et al., 2004), Sigma (Rosenbloom et al., 2016), CLARION (Sun, 2006), ICARUS (Choi and Langley, 2018), and the iCub (Vernon et al., 2007) to name a few. A comprehensive review can be found in Langley et al. (2009) and Thórisson and Helgasson (2012).

In particular, for robots engaged in social HRI, the implementation of some of these cognitive abilities—or of them as a whole architecture—has been demonstrated in the context of autonomous social interaction (Adam et al., 2016; McColl et al., 2016), in studies aimed at the employment of joint action (Lemaignan et al., 2017) as well as in studies where the robot is assisting the humans in achieving their goals (Beer et al., 2014).

The architectures proposed have been developed with different goals, ranging from modeling biological or psychological phenomena (e.g., model human performance in cognitive and perceptual tasks—e.g., ACT-R or CLARION), to trying to model more complex cognitive processes, toward human-level intelligence, as in the case of SOAR or ICARUS. Our work places itself within this second category, with the specific goal of developing a cognitive architecture supporting autonomous behavior for a robotic platform involved in interaction with humans.

Rather than relying on existing complete architectures, designed for context-free human-robot interaction, or even in a broader sense for general intelligence we opted for a simpler approach; this decision was made in order to gain more freedom for future expansions of the architecture and to focus on the very basic components necessary to establish an interaction. In this sense, we took a developmental inspiration, focusing on replicating interactive and adaptive capabilities such as those observed in toddlers. The architecture relies on the robot evaluating the affective state of its human peers and their mode of interacting with the robot as factors which determine the robot's own internal emotional condition, and subsequent choice of behavior. Our framework over its various developments was tested on the iCub humanoid robot.

## 3. Our Framework

In light of the considerations reported in the previous section, our framework for the iCub consisted of the following modules and their functionalities:

- Perception module, processing tactile and visual stimuli^1^;
- Action module, tasked with moving iCub's joint groups^2^;
- Adaptation module, active only in the adaptive profile for the robot and in charge of regulating iCub's social need^3^.

### 3.1. Perception Module

The perception module was tasked with processing stimuli from two sensor groups: *tactile stimuli*—the data processed from the skin sensor patches on the iCub on its arms and torso, which carried information about the size of the area that was touched (expressed in number of *taxels*—tactile elements) and the average pressure of the touch (Cannata et al., 2008); and *visual stimuli*—the images coming from iCub's eye camera, jointly analyzed for detecting the presence of a face and extracting the facial expression of the person, as well as for detecting the presence of some of iCub's toys. The module was realized using iCub's middleware libraries (Metta et al., 2008) for processing the data from the skin covers on its torso and arms; as well as using the open-source library OpenFace (Baltrušaitis et al., 2016) for extracting and analyzing the facial features of the caretaker, represented by their facial action units (AUs) (Ekman et al., 1978).

The data from the OpenFace library were analyzed in order to obtain the most salient action units from the detected facial features. We considered as positive-associated AUs smiling and cheek raising with crinkling of the eyes, and as negative-associated AUs brow lowering, nose wrinkling, and raising the upper lip in a snarl. Presence of all positive AUs was classified as “smiling” (presence of just a mouth smile but no positive upper AUs signified a fake smile, and was not classified as “smiling”), presence of only the brow lowering but without additional negative AUs was classified as “contemplating” whereas the presence of all negative AUs signified “frowning.” If neither of these AUs groups were present in the frame, the user's affective expression was classified as “neutral.”

Our perception module did not assign a positive or negative emotional state and it did not involve training an additional model from the observed action units; rather, it detected and provided as output the appearance of the abovementioned action units. The detected action units signified the person was exhibiting one of the selected facial expressions, which is an approach often used in studies where the perceptual component in the architecture is focused only on facial expressions, and not on more detailed emotional state (Yalcin and DiPaola, 2018; DiPaola and Yalçin, 2019).

**Figure 1:**
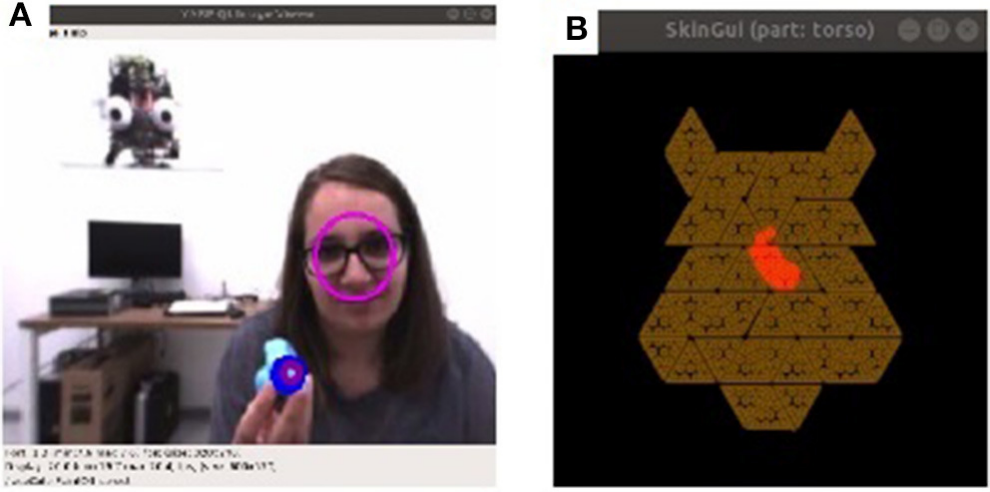
The two outputs from the *perception* module. **(A)** Shows an example of face detection and toy tracking. **(B)** Shows in red the area of the iCub's torso touched by the participant. (Informed consent of participants has been obtained for the use of their photo).

In addition to the affect detection functionality, the visual perception consisted also of the color detection functionality, which was able to detect and track a set of predefined colors, looking for contours in the image of a certain size (fitting the size of the toys) and color. Figure 1A shows the simultaneous detection and tracking of the face of the participant and a toy—the center of the face is indicated with a pink circle, the center of the object with a blue one, and the smaller purple circle instead indicates where iCub's attention is at that moment, i.e., which stimuli it is tracking. Figure 1B instead shows detected touch on the tactile covers of iCub's torso. For the skin there was some additional processing post-extraction; this was because during prolonged interaction, the tactile sensors tended to overheat and give phantom signals. Thus, the data were filtered to register as touch only areas that were larger than 5 taxels and recorded average pressure larger than 12.0. These values resulted from previous experimental exploration of parameters for the skin sensors, as during the overheating the sensors would register small pressure points under 5 taxels when there was no actual touch. These data were processed for the torso and both arms separately, and sent to the perception module.

### 3.2. Action Module

The action module communicated with iCub's middleware (Parmiggiani et al., 2012) and performed a finite set of actions by controlling the specific body part in the joint space. iCub was holding a box of its toys and it could *move its arms* in an extending or flexing motion, thus bringing the box closer to the person or away from them. A *looking* action was performed every time iCub was changing its gaze focus, utilizing motions of the neck and saccadic motions of the eyes. When iCub wanted to engage with the caretaker, it would *straighten up* and look for the person, and then during the interaction engage in gaze-cueing and looking at objects, whereas when iCub was oversaturated and wanted to disengage, it would *lean down* to the table and away from the person, and look down to its toys, ignoring other attempts to engage.

### 3.3. Adaptation Module

This module maintained iCub's comfort and guided the adaptation process. In our framework iCub's comfort was dependent on the amount of social interaction the robot was involved in and the motivation in our architecture was represented by iCub's striving to maintain it at an optimal level. The comfort of iCub grew when a person was interacting with it, whereas lack of any stimuli caused the comfort value to decay. iCub's social architecture was also equipped with a *saturation* and a *critical* threshold, which were reached when the interaction was too intense or was too sparse, respectively.

At the beginning of the interaction with each user, iCub started with its comfort set at 50% of the maximum value. Then the comfort level was updated continuously at the beginning of each cycle of the control loop of the interaction^4^. This happened in the following manner:


if (F[t] || T[t]):
    C[t] = (F[t]+T[t]+C[t-1]τ)/(τ+0.1)
else:
    C[t] = β*C[t-1]


where *C[t]* indicates the current comfort level whereas *C[t-1]* is the previous comfort level; *F[t]* and *T[t]* are the input stimuli from the visual and tactile sensors, respectively. β and τ are the social variables dictating the decay and growth rate of the comfort value, where their initial values were set at β = 0.998 and τ = 500.

When there was a human interacting with the robot (iCub was perceiving a face in front of it, or registering touch with its skin), the comfort *C[t]* at time *t* was updated using the first formula, which takes into consideration both modalities in which the user could interact with iCub, as well as the previous level of comfort *C[t-1]*; on the other hand if iCub was not currently engaged in interaction, its comfort was updated as depicted in the second formula, which calculated the decay of the comfort. This formulation implies that a multimodal interaction (receiving both visual and tactile stimuli) or a longer, steadier interaction increased the comfort at a faster rate.

The variables τ and β were the growth and decay rates, respectively. τ modulated how much *C[t-1]* was taken into consideration: a smaller τ represented a more rapid growth of the comfort when stimuli were detected, and a larger value a slower, steadier growth. β was indicating how quickly *C[t]* decayed without stimuli; the smaller the value of β, the more drastic the decay of the comfort.

If the robot did not receive enough stimulation for a certain period of time or if it was stimulated too much, the comfort could reach either a *critical* or a *saturation* threshold. When one of these threshold values was hit the system adapted. More precisely, the adaptation process had the following pattern:

- If the comfort reached the *saturation* limit (i.e. it exceeded 75% of the total comfort value) this signified that the robot was overstimulated. To cope with this, the robot entered a suspension period of 20 s where it ignored all stimuli and this led to the comfort value going back to the optimal zone. Moreover, to adapt to future interactions with a very interactive partner, the robot increased the value of τ by 500. This implied that in the next very intense interaction, *saturation* would have happened later.
- If the comfort dropped to the *critical* level (i.e. it dropped under 25% of the total comfort value) this signified that the robot had been left alone for too long. As an immediate response the robot attempted to engage the caretaker by calling for attention. If ignored the robot would then enter a 20 s suspension period, simulate stimuli to itself so as to recover back to the optimal comfort level and then adapt. In particular, it increased the value of β by 0.005, in practice increasing the time before it would have hit again the *critical* threshold and ask for attention.

An example of the changes of comfort value during an interaction involving some threshold hits and the consequent adaptation of decay rate is reported in **Figure 4B** and will be described in detail in section 5.1.

To provide a reference of the framework's dynamics—the initial values of the comfort variable, β, and τ provided for 1.5 min of extreme interaction before hitting a threshold (1.5 min of zero stimuli for a *critical* threshold, and 1.5 min of full multimodal interaction for *saturation*). The time limits increased after each architecture adaptation (i.e., threshold hit), e.g., after 2 adaptations prompted by *critical* triggers, iCub could be left by itself for 7.5 min before hitting the threshold again.

The selection of the initial values of τ and β, of the amount of changes in the parameters during adaptation, of the duration of the suspension period and of the *critical* and *saturation* thresholds was based on a previous simulation study (Tanevska et al., 2019): this explored more in depth the impact of each of these parameters on the behavior of the architecture.

The manner of modifying the τ and β variables was inspired from the related research done in Hiolle et al. (2012, 2014). In addition, we implemented the suspension period following adaptation. Originally the architecture adapted by immediately resetting the comfort level back to the optimal level and continuing with the interaction. The suspension period was included as a factor only after the validation of the original architecture with participants, during which it was realized that a continuation of responsiveness of the robot might not have allowed for the participants to infer that they were doing something not ideal for the robot. For example—in the case of *saturation*, after the instantaneous robot withdrawal, it was immediately ready again to respond, which induced participants again to continue to interact in the same manner and trigger again *saturation*.

## 4. Materials and Methods

In this study we were interested in exploring whether adaptation is a necessary functionality for human-robot interaction, and particularly for the context of free-form social human-robot interaction. In a scenario where there would not be a clear task for the human to perform with the robot, would the adaptive functionality bring anything additional to the interaction? To address this, we formulated three related questions:

- How much would the adaptive architecture change for each participant during the interaction, and how would people react to such personalization? (This will be answered in section 5.1)
- What would be the subjective evaluation of the participants for the interaction, and would it depend on the adaptivity of the robot? (This will be answered in section 5.2)
- Would participants change their way of interaction across modalities or robot adaptivity level? (This will be answered in section 5.3).

### 4.1. Experimental Design

A previous exploratory study illustrated that a screen-based game interaction scenario did not provide a desired amount of affective expressiveness in participants (Tanevska et al., 2018a). Since we had explored the effectiveness of a care-taking scenario in prior pilot and validation studies with the iCub robot (Tanevska et al., 2019), we decided to continue in the same direction and expand the existing experimental setup.

The interaction scenario placed iCub in the role of a toddler exploring and playing with its toys, while the participants were tasked as the iCub's caretaker. In the study in Tanevska et al. (2019) we investigated the preference of participants for an adaptive dynamic robotic profile over a static scripted one. We now placed the focus on a different task—evaluating in greater detail the effect of the adaptation modality on the interaction in two otherwise equally dynamic and responsive behavior profiles. In that direction, the two different “personalities” of iCub were both equipped with the full cognitive architecture described in the previous section, with the only difference being that one profile had the adaptation functionality disabled.

In both behavior profiles iCub's behavior was guided by its social skills, and in both conditions iCub began the interaction with the optimal values of the growth and decay variables as selected after the simulation study (Tanevska et al., 2019). The only variation in the profiles was that in the *Fixed* profile (F) the values of τ and β remained unchanged throughout the interaction (regardless of how many times the comfort value hit the *critical* or *saturation* thresholds), whereas in the *Adaptive* profile (A) there was the personalization of the architecture to each participant by modifying the values after each threshold hit.

The interaction between iCub and the participants was mostly free-form; and while iCub could try during the session to also initiate interaction, or would actively ask for it after hitting a *critical* or *saturation* point; for the most part participants had the liberty of guiding the interaction. During the entire interaction iCub could receive and process stimuli from the participants which could be tactile (contact with the skin patches on iCub's arms and torso) and visual (either observing the participant's face at an interacting distance and evaluating the facial expressions, or detecting toys by recognizing their color and shape).

### 4.2. Participants

Twenty-six participants in total took part of the caretaker study. The youngest participant was aged 18 and the eldest 58, with the average age being 32.6 years (SD = 11.98). The gender ratio between the participants was 15:10:1 (M:F:NBGQ^5^).

### 4.3. Protocol

Participants were evenly distributed in two groups of 13 people, where one group interacted first in the adaptive and then in the fixed dynamic setting, and the other vice versa. A session of interaction in either profile setting lasted 12 min, divided in three phases of 4 min, the middle one of which was the interval when participants were asked to work on a secondary task—the pollinator puzzle (see section 4.4).

Between the two sessions of interaction, as well as at the beginning and end of the interaction participants answered questionnaires (more details on the questionnaires in the following subsection), bringing the total time of commitment for the participants at around 45–50 min. There were two environments in which the participants were stationed during their visit—the office setting and the laboratory setting.

Upon their arrival to the institute, participants were first brought to the office setting, where they were presented with the consent form and given time to read through it and sign it. Then, while still in the office, we informed participants that during the experiment there would be several moments during which they would be given different forms of questionnaires—related to their personality, relationship to iCub, as well as creativity and problem solving. This was followed by the familiarization phase for the pollinator puzzle. The concept and rules of the puzzle were explained to the participants, and they were presented with the first pollinator puzzle (the purpose of which was to obtain the baseline for each participant's performance). The participants were timed for 4 min (the amount of time allotted for the puzzle during the familiarization phase was the same as the time during the robot interaction). After the time ran out (or if participants completed the puzzle in less time—after they were done), we escorted the participants from the office and took them to the laboratory.

On the way to the laboratory we briefed participants on the experiment. More specifically, they were told that they would have roughly half an hour of free interaction with the robot iCub Reddy, who is equipped with a toddler-like personality. We informed them of the modalities they could interact on with iCub, albeit in an informal way – “iCub can see you, it^6^ can feel you when you pet it, it likes hearing you talk to it even though it does not understand you, it speaks its own language.” Participants were purposefully informed that iCub likes hearing them because it was observed in our previous pilot study that people who knew iCub was not capable of speech recognition did not talk at all to the robot during the study.

**Figure 2:**
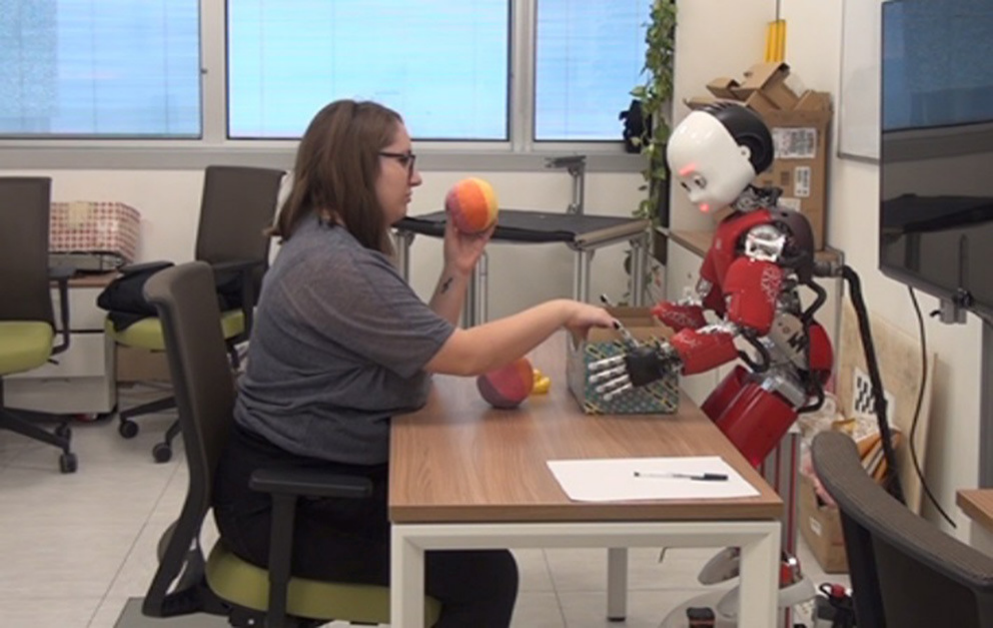
The layout of the laboratory setup. Informed consent of participants has been obtained for the use of their photo.

In the laboratory iCub was positioned in front of a table and holding a box with toys (as shown in Figure 2), some of which were out of the box and spread across the table at the beginning of the interaction. The participants were offered a chair in front of the table facing iCub, but they also had the freedom to sit or walk anywhere in the room.

When iCub was in a state of interacting with its caretaker, it maintained mutual gaze and tracked the person's face, or if the person was playing around with some of the toys it would track the toy that was nearest to it. If the person was not showing any toys to iCub, it would occasionally break mutual gaze and try to indicate toys to the person by looking down at a toy and back to the person (gaze-cueing), by saying the name of the toy or by moving the box toward the participant. In order to avoid giving participants the impression that iCub could understand them, the verbal utterings (which were the names of the colors iCub could recognize, as well as some encouraging and protesting sounds in order to attract attention or to disengage) were recorded in Macedonian (the native language of author A.T.) and then processed and low-pass filtered so as to both make them sound more robotic as well as unintelligible to participants.

Additionally participants were reassured that any perceived lack of interest or reciprocity on iCub's part was due to the robot switching its attention to something else (in line with its toddler personality), and not due to them interacting “in a wrong way.” This was also deemed necessary to be included in the protocol due to a similar realization from the previous pilot that some people were getting worried when iCub would switch its attention and they thought they “did something wrong.”

### 4.4. Secondary Task

With the goal of further exploring the potential benefits of having *critical* and *saturation* thresholds in the architecture, we devised an approach to manipulate the behavior of the participants by introducing a timed secondary task at a certain point in the interaction. This task was designed to observe changes to the interaction patterns if participants were suddenly given a secondary task, but the robot was still asking for their attention.

For this, a task needed to be considered that would involve a cognitive load on the participants, while at the same time being a task that would neither be too time-consuming (like sudoku), nor too attention-demanding or distracting (like a phone call during which participants would be tasked to write down some information). The solution selected was to present participants with some easier mathematical problems involving the basic arithmetic operations; this meant finding a set of numeric puzzles that would be both simple enough to do in a short time interval, but also appealing and interesting. The final choice for the secondary task was the pollinator puzzle^7^.

**Figure 3:**
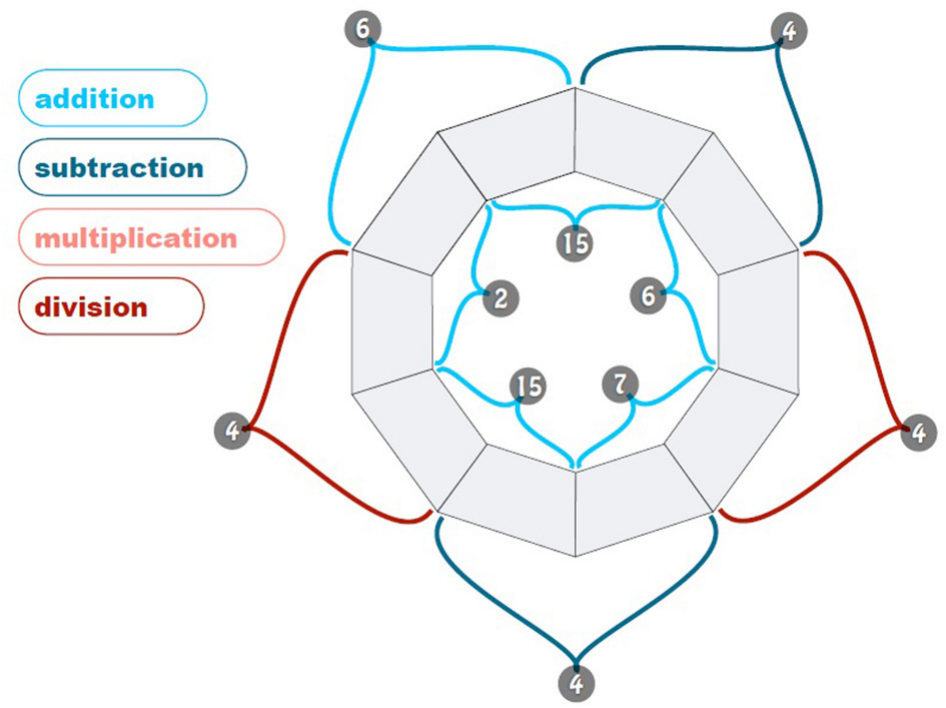
Sample pollinator puzzle.

The pollinator puzzle is a logic-based, combinatorial number-placement puzzle, where 10 empty fields are arranged in a flower-like shape (see Figure 3). The digits 0–9 need to be placed in the empty fields, each digit appearing one time only without repetitions, in such a way that each pair of digits gives the specified result for the operation on the petals. Each puzzle has only one possible solution following these rules.

### 4.5. Data Analysis

We collected data from four main sources during the study—the questionnaires filled by the users; the evaluations of the filled pollinator puzzles; the video and audio recordings from the external camera; and the data collected by the robot during the interaction phases from the tactile sensors, internal camera, and state machine output.

#### 4.5.1. Questionnaires

Participants responded to questionnaires at three points during the interaction study. The first set of questionnaires was done after they entered the lab with the robot but before beginning with the interaction, the second set was halfway through the interaction (which in reality was the moment after which the robot switched personalities, unbeknownst to the participants), and the last set was at the end of the interaction.These three points in the interaction are labeled as PRE, BETWEEN, and POST in the Results.

All three sets of questionnaires collected the IOS rating of closeness between participants and the robot (Aron et al., 1992), as well as the Godspeed questionnaires on animacy and likeability (Bartneck et al., 2009). Additionally, in the second and third set of questionnaires there was also a qualitative open question asking participants to describe the interaction using three adjectives, as well as a set of questions related to how they perceived the interaction with the robot. Finally in the third set of questionnaires there were two descriptive questions related to the different sessions, and the TIPI questionnaire.

#### 4.5.2. Pollinator Puzzle

Participants did in total three rounds of the pollinator puzzle. One was completed as a baseline before starting their interaction with the robot, one during the first interaction session and one during the second interaction session. There were two evaluation metrics for the puzzles—the % of filled fields (out of the 10 empty fields) and the % of accurately filled fields (out of the 10 empty fields).

A combination metric was then designed in order to obtain a single evaluation value, where if X was the percentage of completeness and Y the percentage of accuracy, the final metric Z was obtained as Z = 0.4*X + 0.6*Y. The combination metric was designed with the goal of taking into account as factors both the accuracy and the completeness, but give a higher reward for the accuracy.

#### 4.5.3. Internal Data From iCub

From the iCub itself we recorded the tactile and visual data, as well as all of the values of the architecture—the fluctuations of the comfort value and the changes to the decay and growth rates. The data from the architecture was annotated for each frame received by the robot with a timestamp and the state (of the state machine) that iCub was in.

## 5. Results

### 5.1. Architecture Dynamics

The cognitive framework developed for iCub was a continuously-changing one, learning by modifying its social variables and adapting to the person's frequency and intensity of interaction. This means that the ways in which someone interacted with the robot provoked changes in the internal states of iCub and its comfort level. Every time a threshold of the robot's comfort was hit, iCub adapted the appropriate comfort variable and changed its behavior accordingly.

If the *critical* threshold was hit, signifying lack of stable interaction with the person, iCub modified its decay rate and as a result could remain in an idle state for longer periods of time before it would need again to interact with the person. On the other hand, hitting the *saturation* threshold meant iCub was engaged with a person who was more intense in the way it behaved and interacted with iCub (using multiple modalities and interacting for a long stable period of time), so iCub modified its growth rate which enabled it to stay interacting for longer time.

**Figure 4:**
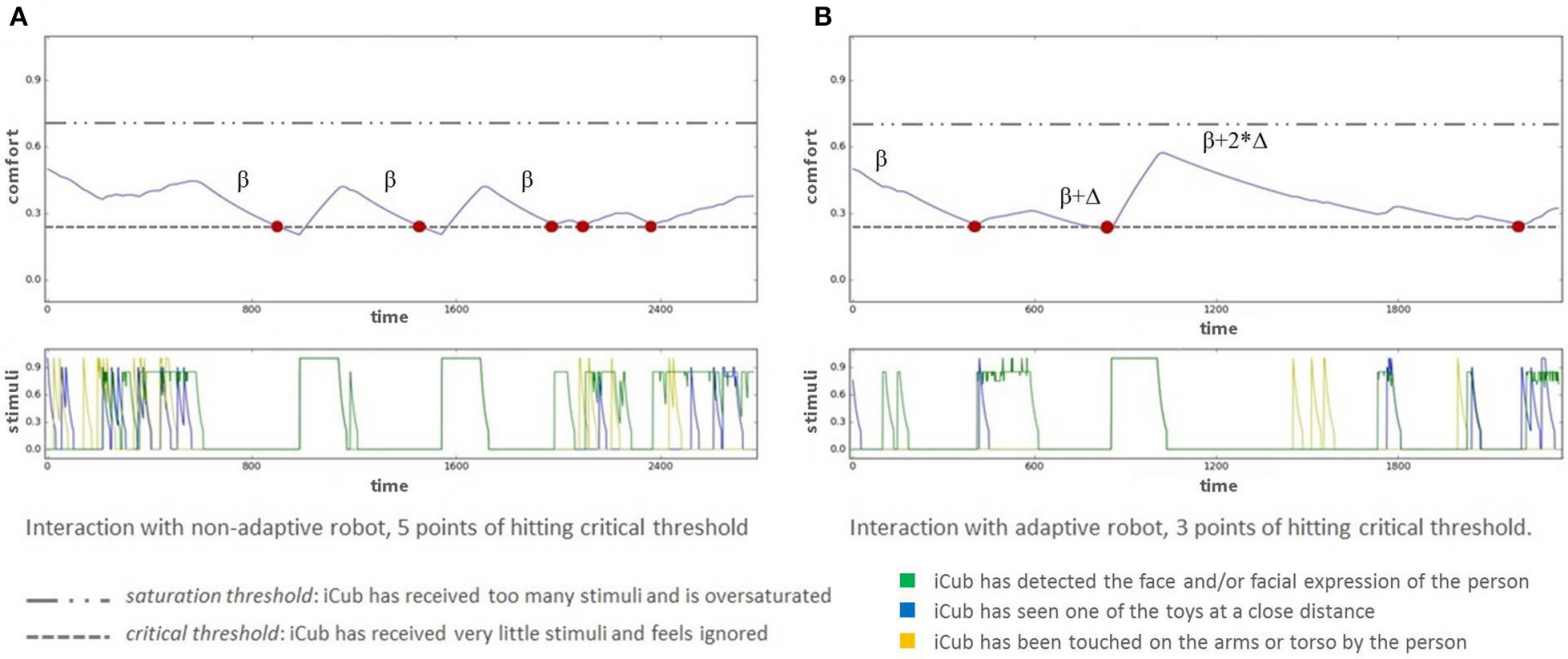
Architecture dynamics: upper graphs depict the variations in iCub's comfort value over the course of an interaction session, lower graphs depict the occurrence of stimuli. *Critical* hits are shown in red dots. **(A)** Sample FA participant interacting in the first Fixed session. The comfort value hits 5 times *critical* threshold, but the decay rate doesn't change. **(B)** Sample AF participant interacting in the first Adaptive session. The comfort value hits three times the *critical* threshold. After each hit the decay value changes.

Figure 4 shows the behavior of the architecture and the flow of iCub's comfort value for two different participants in different sessions of interaction. Figure 4A illustrates the behavior of the architecture for a participant that had its first interaction with the robot in the Fixed session. Here the critical threshold was hit first two times while the participant was performing the secondary task, and the participant ignored the robot's attempts to engage; and additional three times in the last phase of the session after the timer for the secondary task ran out, but in these three instances the participant was no longer distracted and answered iCub's calls.

There are two reasons why the three responded calls are so close in succession one after the other. The first reason is that iCub was in the Fixed personality, so it did not adapt to the person's reduced interaction during the secondary task. This explains why the first three (out of the five in total) threshold hits happened at an identical regular period. The last two threshold hits instead happen so close to each other since the participant responded unstably to iCub's calls, in a manner of giving brief stimuli and then turning their attention to something else, which did not provide iCub with enough stability to be comforted. Instead in the final instance when the *critical* threshold is hit the participant's response was a more stable one, interacting on several modalities, so as a result iCub's comfort resumed growing.

Figure 4B, on the other hand, shows the interaction between iCub and a participant interacting with it for the first time in the Adaptive session. This participant was less interactive than the participant in Figure 4A, but even so the total number of threshold hits was three, out of which only one was not answered. This demonstrates the effectiveness of the adaptivity of the architecture, which can be observed also in the decay slope during the secondary task. After two adaptations of the architecture, the decay slope is much slower, preventing iCub from hitting another *critical* point until very near the end of the interaction.

Regardless of the order of the interaction sessions (AF or FA—standing for Adaptive-Fixed and Fixed-Adaptive, respectively) or the phase of interaction, overall during the experiment on average people hit a threshold on average 1.42 times during one session: on average 1.79 times during the first session and 1.04 times during the second.

The total number of threshold hits summed for all participants was 68, out of which only 2 (3%) were *saturation* hits, and all remaining ones (97%) were *critical*. In these calculations the first two participants were excluded due to technical reasons rendering their number of threshold hits unusable.

**Figure 5:**
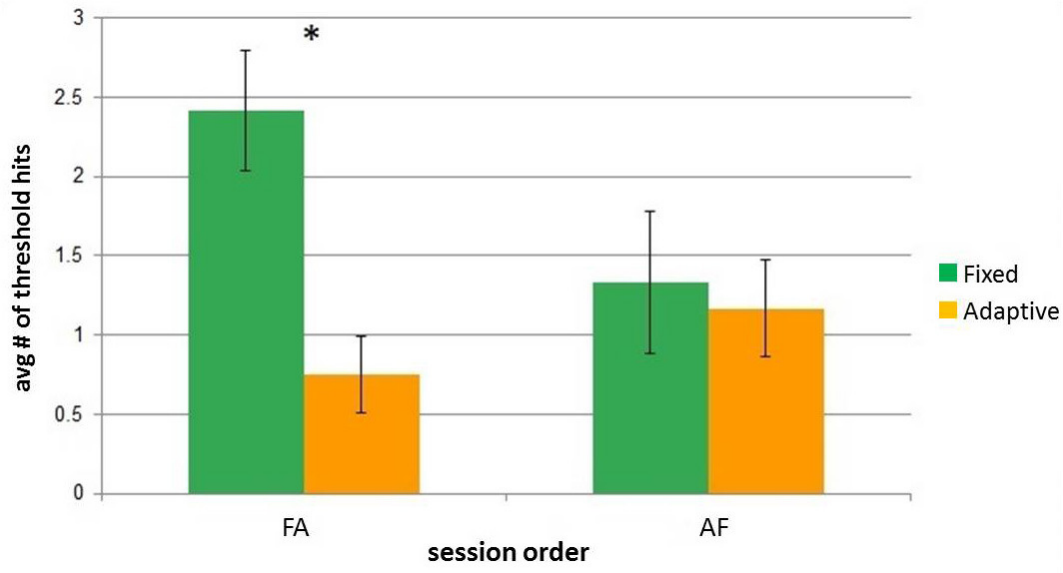
Comparison of average amount of threshold hits per session and order group. Error bars represent standard error. Asterisks indicate significant difference.

Figure 5 illustrates the effect of the order of the sessions on people's first interaction with iCub. Overall, the participants in the FA group had noticeably more threshold hits in the Fixed session than in the Adaptive, whereas the participants in the AF group had a roughly similar ratio of total threshold hits in the Fixed and Adaptive sessions.

This was additionally confirmed after running a mixed-model 2-factor ANOVA, with SESSION (levels: adaptive and fixed) and ORDER (levels: AF,FA; signifying the groups of participants) as the within and between factors, respectively.

A significant difference was found both over the SESSIONS [*F*_(1, 22)_ = 7.87, *p* = 0.01] and for the interaction [*F*_(1, 22)_ = 5.27, *p* = 0.03]. The ORDER had no significant impact [*F*_(1, 22)_ = 0.68, *p* = 0.42]. A *post-hoc* Bonferroni test confirmed that the number of hits was significantly larger in the Fixed session, for the group who encountered that as first one (FA group).

**Figure 6:**
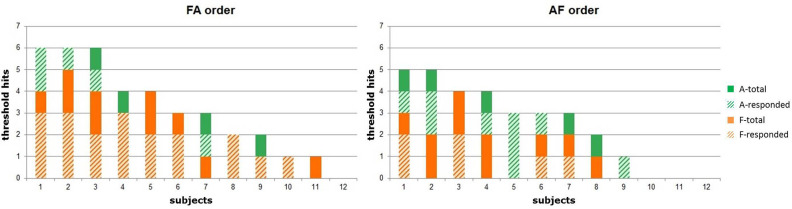
Number of occurred and responded threshold hits per session for FA and AF participants.

A deeper analysis into the individual modes of behavior are presented in Figure 6. This analysis consisted of measuring the changes in the architecture for each participant, comparing for the two different orders of sessions how many times the thresholds of the architecture were hit, as well as how many times people responded to the calls for interaction at the *critical* threshold.

While a large variety in the number of threshold hits (ranging from 0 to 6) can be seen in both conditions and across both session orders, it can be noticed that the majority of people showed a tendency to respond to the robot's calls. Some participants never hit a *critical* or *saturation* threshold (indicated at the end of both figures), however there were only two participants who did not respond to the robot's calls for engagement, suggesting that in addition to iCub being adaptive in some cases, participants always adapted to the robot.

The analysis of the architecture dynamics highlights the differences between the sessions, depending on which was the starting session for participants, as shown in Figure 5. Fixed-Adaptive participants had a more challenging first session since it was both the first session of interaction with the robot, and the session where the architecture did not adapt to their interaction particularities. On the other hand, the Adaptive-Fixed participants' first session of interaction with the robot was the one in which iCub was adapting its comfort variables to their interaction profiles; this contributed to them having less threshold hits in their Fixed session when compared to their FA counterparts.

### 5.2. Subjective Evaluation

**Figure 7:**
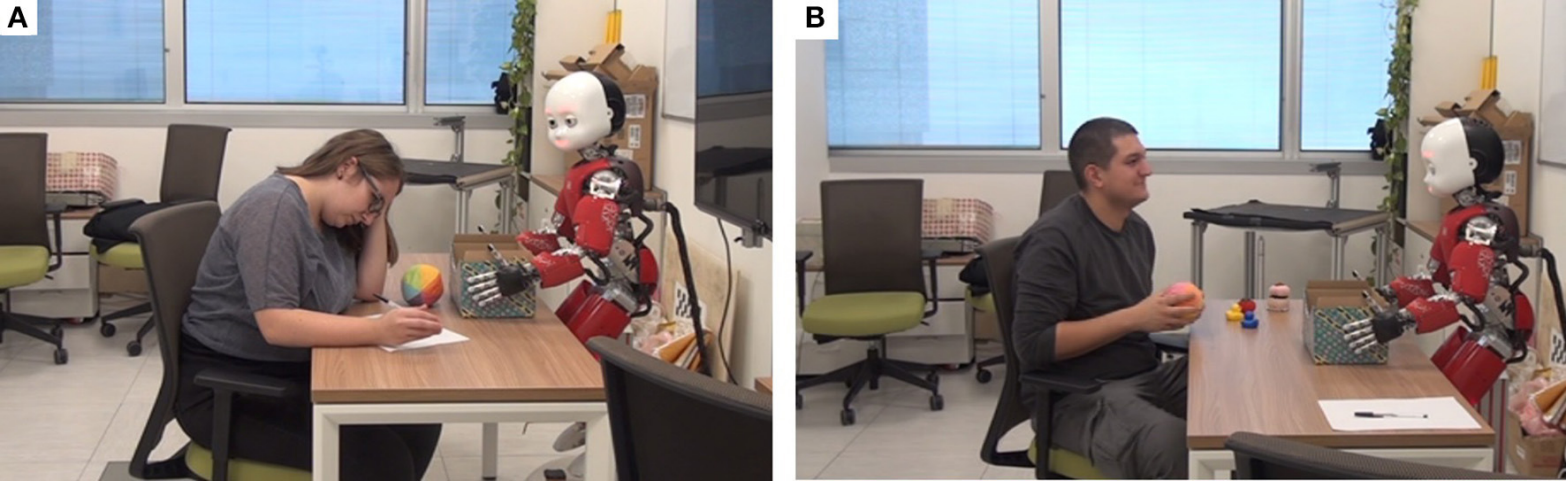
Participants in interaction with iCub. **(A)** Participant working on the pollinator puzzle. **(B)** Participant interacting with iCub. Informed consent of participants has been obtained for the use of their photo.

The subjective evaluation included exploring the expressed preference of participants for interacting with iCub in the Adaptive or Fixed session, their ability to differentiate between the two different profiles of the robot, and evaluating whether their IOS and Godspeed ratings changed as a function of the time spent with the robot or the adaptivity of the robot. Figure 7 depicts participants in different phases of interaction with iCub. Before analyzing the data from the questionnaires we verified their reliability using Cronbach's Alpha, which yielded a reliability coefficient of 0.9.

**Figure 8:**
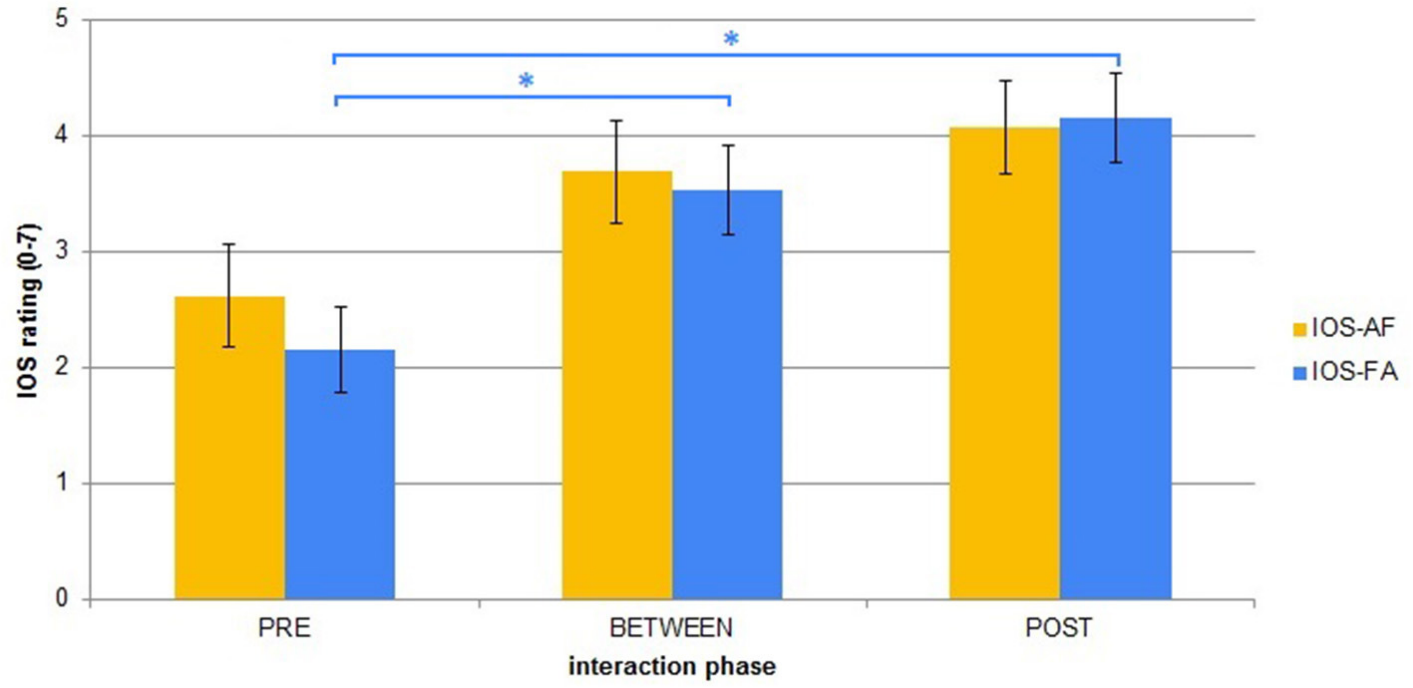
IOS ratings averages across order and phases. Error bars represent standard error. Asterisks indicate significant difference.

**Figure 9:**
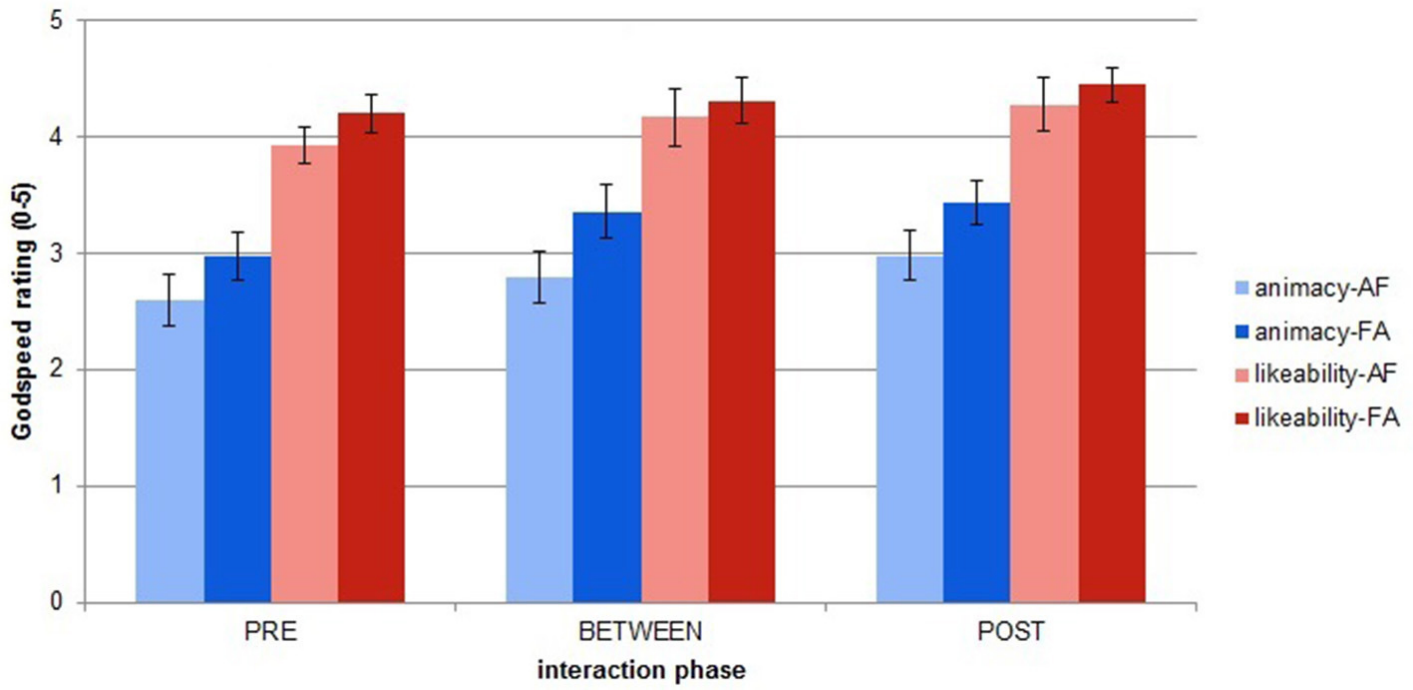
Godspeed ratings averages across order and phases. Error bars represent standard error.

In this study, we wanted to explore the comparison between two similarly dynamic and responsive architectures, where the only difference between them was the inclusion of the adaptive component. We were curious to investigate the effect on the adaptivity level of iCub to the participants' self-rated feelings of closeness with the robot (the IOS rating) and the participants' evaluation of the robot's animacy and likeability (the Godspeed ratings). Figures 8, 9 show the average of the participants' IOS and Godspeed evaluations before interacting (PRE), between the two interaction sessions (BETWEEN), and at the end of interaction (POST).

A Lilliefors test for normality indicated that the data were distributed non-normally (all *p*'s < 0.01), so a statistical nonparametric analysis was performed on all IOS and Godspeed ratings. To check the variations between the three different phases of rating (PRE experiment, BETWEEN sessions, and POST experiment) we ran three Friedman ANOVA tests.

Participants were consistent in their Godspeed Likeability and Animacy ratings, and the Friedman ANOVA tests found significant differences only for the IOS ratings for both the AF [χ^2^_(13, 2)_ = 10.67, *p* < 0.01] and FA group [χ^2^_(13, 2)_ = 16.47, *p* < 0.01], however the *post-ho*c analysis with a Wilcoxon signed rank test showed that the only significant difference after Bonferroni correction was for the FA group between the PRE and BETWEEN ratings (*p* = 0.015) and PRE and POST ratings (*p* = 0.013).

From this analysis what we observed was that participants' rating of their perceived closeness with iCub changed modestly as a result of them spending more time in interaction with it, but not as a function of the adaptivity of the robot. This could signify that on their part, participants did not perceive any structural difference between the two sessions. This could be explained by the assumption that the two sessions were not particularly different to the participants, who did not exploit the adaptivity of iCub excessively. Alternately, notwithstanding the differences experienced by the participants, both sessions could have been equally “likable” to them.

While people seemed more consistent in their Godspeed ratings across all sessions, their IOS ratings tended to be more variable, with bigger differences (usually of 1, but also reaching 2 and 3) between the different sessions. However, the same conclusion was also evident here; the rating of IOS closeness increased for most people as a consequence of the prolonged time spent with the robot, and not as a result of the robot's adaptability. It would seem that although there were differences in the two sessions, people did not change their rating.

This was confirmed also by the free questions they had to answer after the second session:

- Which session did you prefer and why?
- What was the difference (if any) you noticed between the two sessions of interaction?

Seventy-seven percent of participants answered that they preferred the second session because they felt iCub was more animated or interactive toward them, 19% replied that they enjoyed both sessions equally and only one participant said he did not enjoy any of the two. Additionally, 27% answered that they did not perceive any difference between the sessions, 46% instead had perceived the robot being more interactive in the second session (however from those 46% half were FA and half AF, signifying random chance), and 23% said they learned how to interact better in the second session.

### 5.3. Behavioral Evaluation

After analyzing the subjective evaluation, the final step was processing the behavioral results, which measured how the interaction between iCub and the participants actually unfolded. The behavioral evaluation of the participants analyzed whether people actually interacted differently with the robot across different phases and modalities. This was considered again as a function of the time spent with the robot or the session order (Adaptive-Fixed or Fixed-Adaptive). An additional analysis was done on into how the participants' behavior changed during the dual task.

**Figure 10:**
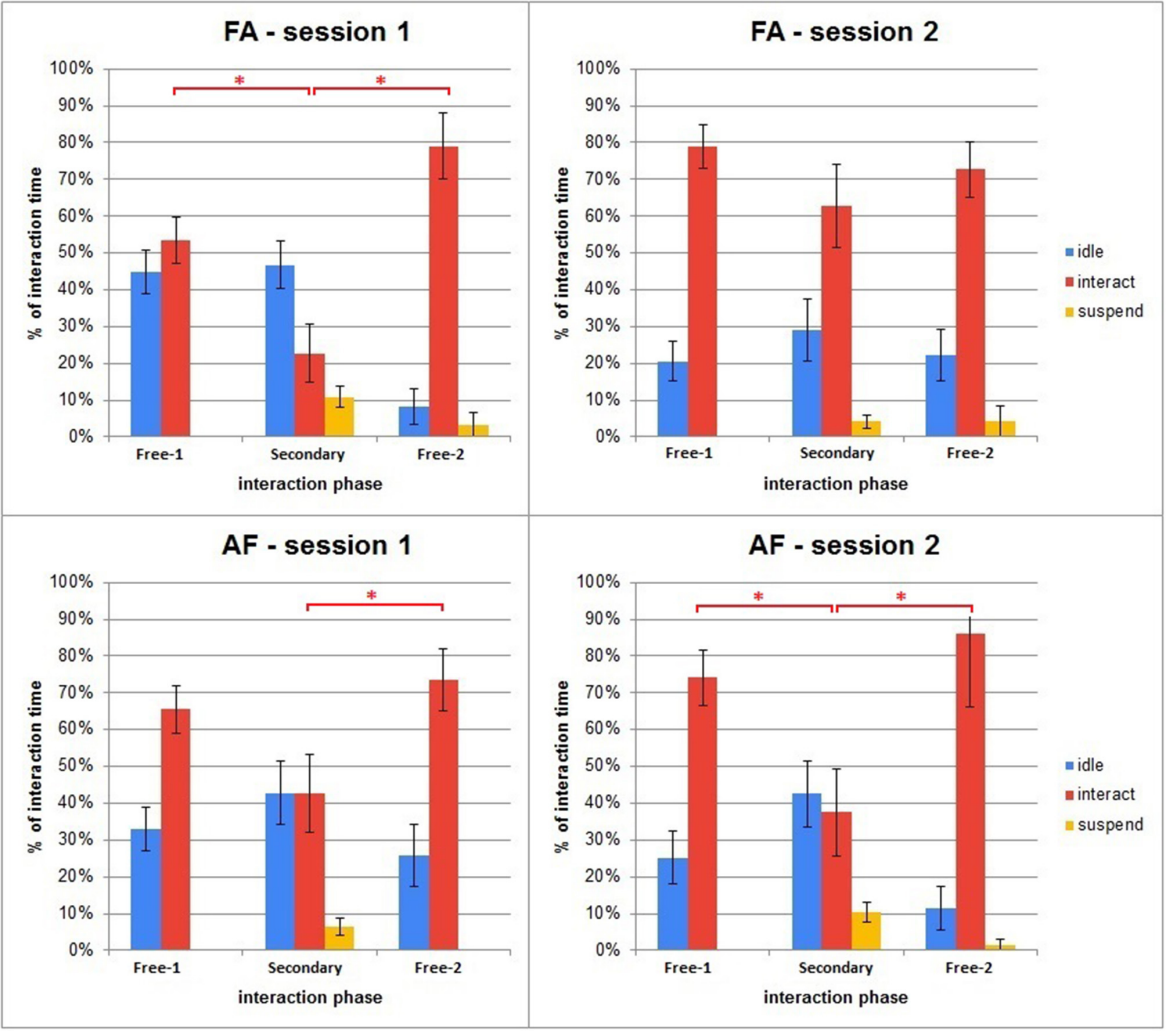
Average percent of interaction times spent in the major states, shown across phases and sessions. Error bars represent standard error. Asterisks indicate significant difference.

This section covers the results from the different modalities of interaction—i.e., how people interacted with iCub on the three modalities of visual-face, visual-objects (also mentioned under visual-toys), and tactile; the distribution of iCub's states during the interaction and all three phases for each session, and finally how the secondary task impacted the interaction. Figure 10 shows the distribution of the states the robot was in during the interaction.

During the interaction sessions, iCub's behavior was guided by a state machine. The three main states were *idle*, when the robot was left without stimuli from the user and interacted by itself; *interact* when engagement had happened by either party; and *suspend* which iCub entered after hitting a threshold hit and its call for engagement was not responded to (for *critical* hits). There were also more minor, transitional states signaling a change in behavior or an occurrence of the architecture adapting. However since these lasted only a few frames (and in real interaction time, <3 s), they were not taken into account.

From Figure 10 several conclusions can be obtained:

- The interaction pattern among the different phases was similar between the two groups;
- In the third phase (the interaction after the secondary task) there seems to be compensation for having previously ignored the robot, in the form of increased interaction. This can be seen especially in the Fixed session (regardless of order group), potentially because the robot asked for more attention without adapting to the users ignoring it;
- The distribution pattern of the states in the last phase of the first session tends to carry over to the first phase of the second session, indicating participants learning how to interact effectively with the robot. This effect was particularly not expected for the participants of the AF group, since in their second session of interaction the architecture values of the robot were reset, thus, the AF participants essentially interacted first with a robot that adapted to them, and then with one that had no adapted specifics;
- The interactive behavior during the dual task seems to change between the two sessions for the FA participants. Having ignored the robot during the secondary task in the first session (Fixed) where it did not adapt to them, they seem to overcompensate in the secondary task in the second session (Adaptive). As a result, there is a huge jump in interactivity. This may be a combined effect of both overcompensation combined with the added adaptivity of the robot;
- The interactive behavior during the dual task stays nearly identical for the AF participants between the two sessions. As the robot was adaptive in the first session (Adaptive), it adjusted to them; however, due to them not perceiving the robot as particularly annoying or demanding for attention in their first session, there is no compensation in the second session.

A Lilliefors test for normality indicated that the data were distributed non-normally (*p* < 0.01). To evaluate the difference between the interaction patterns in the different phases and between the different groups, we performed several nonparametric tests. We first ran Mann–Whitney *U*-tests, testing for significant differences in the percentage of interaction time between corresponding phases in the AF and FA groups. Then, for each session, we performed a Friedman ANOVA test, followed with a Wilcoxon signed-rank as *post-hoc*, to evaluate the differences among the different phases in a given session.

The Mann–Whitney tests found no significant difference between the AF and FA groups in any of the phases, signifying that in general the two groups of people did not have significantly different interaction patterns in the different phases. The Friedman ANOVA tests found significant differences for both groups in the Fixed session of interaction [χ^2^_(13, 2)_ = 14.6, *p* < 0.01 for AF group; χ^2^_(13, 2)_ = 14.17, *p* < 0.01 for FA group], and for the AF group in the Adaptive session as well [χ^2^_(13, 2)_ = 6, *p* = 0.049]. The Wilcoxon signed rank for the two Fixed sessions showed significant differences between the 1st and 2nd and 2nd and 3rd phase of interaction [*p* < 0.01], whereas for the Adaptive session for the AF group the difference was significant only between the 2nd and 3rd phase [*p* < 0.01]. All differences are significant after a Bonferroni correction.

Furthermore, we compared the percentage of interaction time during the Secondary phase between the first and the second sessions for both groups. The Wilcoxon signed rank showed no significance for the AF group (*p* = 0.534), whereas for the FA group a significant increase was registered in the Adaptive session (*p* = 0.027), which did not resist Bonferroni correction.

**Figure 11:**
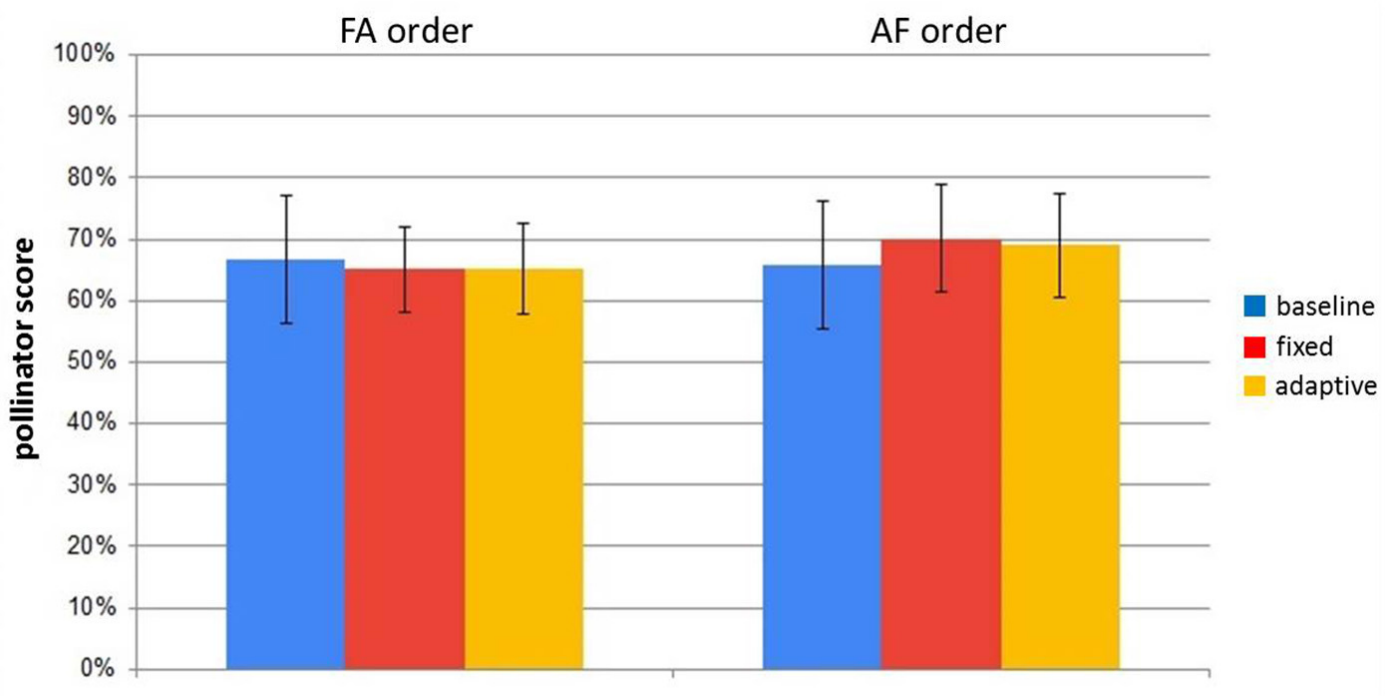
Average pollinator scores for the three times participants did the puzzle. Error bars represent standard error.

Since there was a noticeable difference in how people behaved with the robot while they were tasked with the pollinator puzzle, the next analysis focused on the scores obtained by participants in the pollinator puzzle. From Figure 11, showing the averaged pollinator scores for both groups (AF and FA) over the three times they filled the puzzle—baseline, first session, second session) it can be noticed that there is no difference over the average score. This signifies that even in the phases when the robot was non adaptive, on average participants could complete the task to some extent.

With this analysis it was established that the participants' behavior during the secondary task (interacting with the robot or ignoring it in order to focus on the task) did not strongly impact their pollinator score. In other words, how good people were at the task was subjective for each person, and did not depend on whether they interacted a lot with the robot or ignored it.

**Figure 12:**
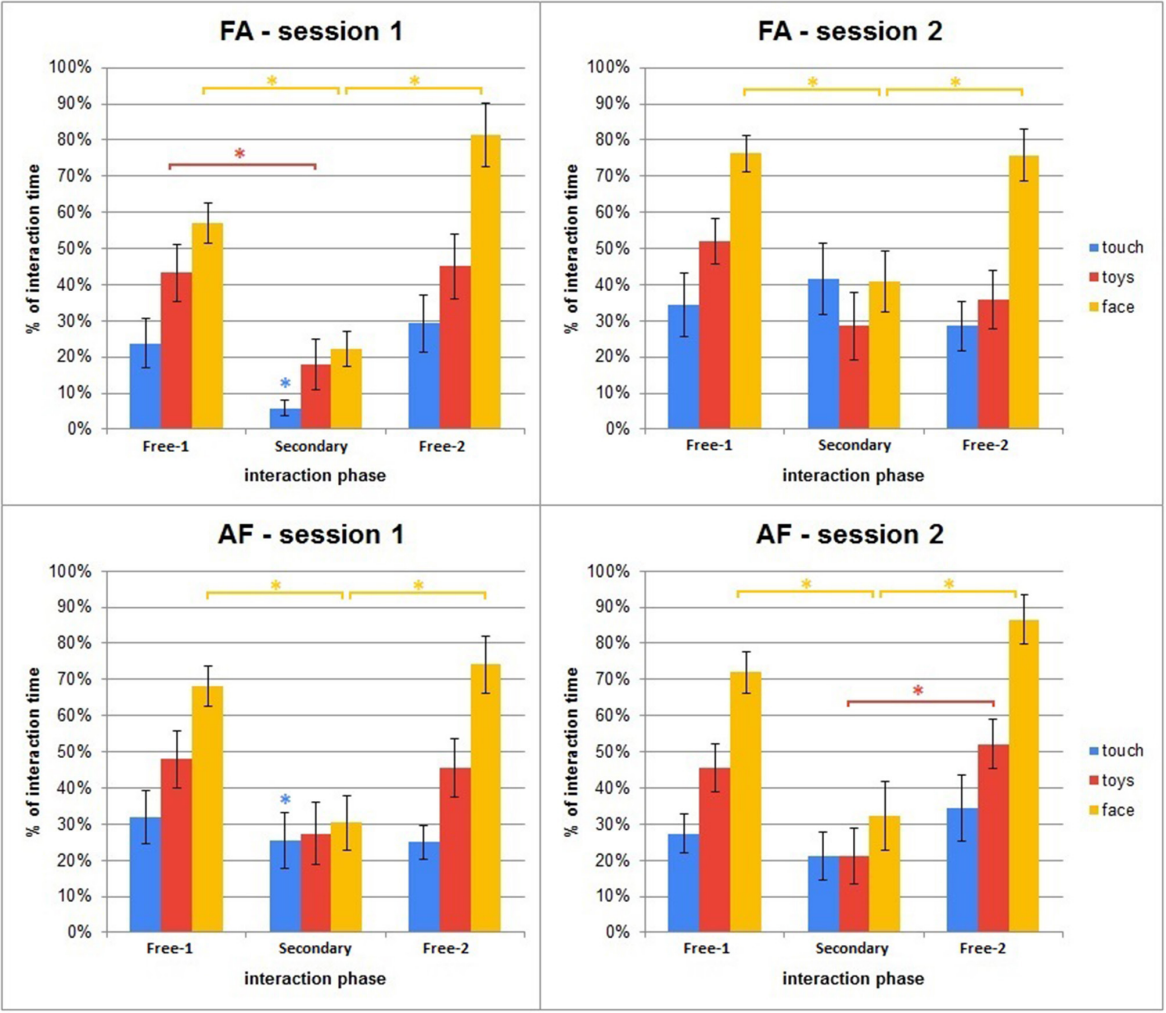
Averages of the perceived stimuli in different modalities during the interaction, shown across phases and sessions. Error bars represent standard error. Asterisks indicate significant difference.

The last step of the analysis looked into the modalities participants used when interacting with iCub. The modalities graphs shown in Figure 12 show that during the secondary task there is, somewhat understandably, the biggest drop in face as input, but there is compensation with touch, which stays similar and does not have such a significant drop. The patterns in the last phase of session 1 tend to be nearly identical to the first phase of session 2, the reason for this could be that the mode of interaction tends to carry over between the two sessions. A similar pattern can be also observed in the analysis of the interactivity distribution in Figure 10.

We next wanted to evaluate the difference between the interaction patterns in the different phases and between the different groups. Since a Lilliefors test indicated that the modalities data were distributed non-normally (*p* < 0.01), we repeated again the same kind of analysis as for the interaction behavior. For each of the three modalities we first ran a Mann–Whitney *U*-test testing for significant differences in the interaction patterns between the AF and FA groups in each phase. We followed that with four Friedman ANOVA tests (one per session) with a Wilcoxon signed-rank test as *post-hoc* to evaluate the differences between each of the phases in a given session.

- Touch: The Mann–Whitney test found a significant difference between the AF and FA groups in the first session of interaction, during the 2nd phase (*p* < 0.01). The Friedman ANOVA tests found no differences between the phases for any group and any session;
- Toys: The Mann–Whitney test found no significance between the AF and FA groups in any of the phases. The Friedman ANOVA tests showed significant differences for the AF group in the Fixed session [χ^2^_(13, 2)_ = 8, *p* = 0.018], and for the FA group in both sessions of interaction [χ^2^_(13, 2)_ = 6.71, *p* = 0.035 for Fixed; χ^2^_(13, 2)_ = 10.17, *p* < 0.01 for Adaptive]. The Wilcoxon signed rank showed significant differences only for the Fixed session of interaction, for the AF group between the 2nd and 3rd phase (*p* < 0.01) and for the FA group between the 1st and 2nd phase (*p* < 0.01). All differences are significant after a Bonferroni correction.
- Face: The Mann–Whitney test found no significance between the AF and FA groups in any of the phases. The Friedman ANOVA tests showed significant differences for both the AF group [χ^2^_(13, 2)_ = 15.8, *p* < 0.01 for Fixed; χ^2^_(13, 2)_ = 14, *p* < 0.01 for Adaptive] and the FA group [χ^2^_(13, 2)_) = 17.76, *p* < 0.01 for Fixed; χ^2^_(13, 2)_ = 7.38, *p* = 0.025 for Adaptive]. The Wilcoxon signed rank showed significant differences between the 1st and 2nd phase, and 2nd and 3rd phase (*p* = 0.01 for AF group, Session 1, between 1st and 2nd phase; *p* < 0.01 for all other tests). All differences are significant after a Bonferroni correction.

Even though the subjective evaluation of the participants did not reveal a correlation between the adaptiveness of the robot with its likability, or an awareness among the participants of the existence of a difference in the profiles at all, there were implicit results pointing to the opposite. The manner of interacting with the robot, both in terms of frequency and use of modalities, changed noticeably, particularly when participants were given the secondary task. More precisely, when the robot was in its adaptive profile, even if the people were given another task to complete, they still managed to interact with the robot in parallel.

## 6. Discussion

Different individuals have different inclinations in how they interact with others, which can also be seen in their approach to interaction with robots. At the same time, different tasks require different levels of human intervention (or the robot requiring help). Creating a unique robot behavior or personality that is able to fit with task constraints and at the same time with individual desires is extremely challenging. Endowing the robot with a possibility to adapt to its partners' preferences is therefore important to grant a certain degree of compliance with individual inclinations.

Our study aimed to tackle this issue by developing a personalized adaptive robot architecture. This architecture enabled the robot to adjust its behavior to suit different interaction profiles, using its internal motivation which guided the robot to engage and disengage from interaction accordingly, while also taking into account the behavior of the person interacting with it.

The caretaker study brought to light two opposing but valuable findings. Participants were not consciously, or at least on an affective level, aware of experiencing two different robotic profiles. When asked explicitly to report a difference between the two sessions of interactions, the majority of participants did not report one, or they reported their feeling that the second session had the more interactive robot profile. This, however, was strongly influenced by the fact that nearly all participants reported that they preferred the second session of interaction, signifying that it was not the profile of the robot that influenced their feeling, but rather the personally gained knowledge on how to better interact with it due to the prolonged time spent in interaction. However, their manner of interacting with the robot showed noticeable changes depending on the phase and session they were in, as well as depending on the robot behavior during the secondary task.

This has several implications, especially for designing different HRI scenarios. This study has addressed free-form interaction and investigated how an adaptive robot could personalize to its caretaker; if we are imagining to port this architecture to an HRI study where the robot would need to learn by processing informations from visual or tactile stimuli, the implications from this study's findings show that the robot would be still capable to receive and process the necessary information from the person, even if the person was not be highly responsive or present at all times.

Additionally, the element of adaptability and personalization in the cognitive framework was not shown to bring any uncertainty and unpredictability. While on a conscious level they remained unaware, the adaptability of the robot still impacted the efficacy of the participants' interaction. Moreover, the presence of the *critical* and *saturation* thresholds promises an another layer of complexity that could be added to the interaction.

A robot that has a *critical* boundary can actively try to initiate interaction with a person, which could be useful not only in scenarios where a person might lose track of the robot or get distracted, but also in scenarios where a person might be very interested to interact with the robot but their shyness might prevent them from attempting to engage the robot first.

Similarly, a *saturation* boundary is not only useful for evaluating how much a person is interested in restarting an interrupted interaction, but can be also a crucial element in multi-person HRI scenarios, or if the robot needs to also accomplish some other task in addition to interacting with the people. The *saturation* threshold in particular was something that did not get used in its full potential in our study, which was probably due to the above-mentioned effects not carrying over to an 1-on-1 HRI scenario.

A limitation of our study can be found in the fact that even though the interaction was designed to be as free-form as possible, it was still a very simplified scenario of interaction. This was also due to the limitations of current state-of-the-art: artificial cognitive agents (such as robots) are not yet at the level of replicating the human cognitive abilities, and the aspect in which this was felt most keenly was in the absence of a verbal interaction between the participants and iCub.

Adaptivity is a very important building block of cognitive interaction. In this way, endowing a humanoid robot like iCub with adaptability, even in a scenario with behavior modalities of lower cognitive intelligence, is already a first step towards approaching personalized and cognitive human-robot interaction. Indeed, this effect can be seen even in children; we readily observe their limited capabilities in the fact that before 2 years of age they are, for the most part, not speech-proficient. However, they are still cognitive agents that are very efficient at establishing adaptive interaction as a function of their partner, be it a peer or a caregiver.

The hope and future direction of this research is that by investigating other cognitive functionalities to implement and testing other scenarios of interaction, the adaptive framework will reach the point of a more individualized, long-term, and generalized interaction between humans and robots.

## Data Availability Statement

The datasets generated for this study are available on request to the corresponding author.

## Ethics Statement

The studies involving human participants were reviewed and approved by Comitato Etico Regione Liguria-Sezione 1. The patients/participants provided their written informed consent to participate in this study. Written informed consent was obtained from the individual(s) for the publication of any potentially identifiable images or data included in this article.

## Author Contributions

All authors contributed to the design of the experiment. AT cured the data collection. AS and AT cured the data analysis. All authors contributed to the writing and revision of the manuscript.

## Conflict of Interest

The authors declare that the research was conducted in the absence of any commercial or financial relationships that could be construed as a potential conflict of interest.

## Footnotes

^1^ https://github.com/TadasBaltrusaitis/OpenFace/wiki

^2^ https://github.com/robotology

^3^The code for the adaptation module is in a private repository, but is available on request.

^4^Referring here to the perception-action control loop of iCub's architecture.

^5^NBGQ—non-binary/genderqueer.

^6^The participants who spoke only Italian were briefed in Italian instead of English. Due to Italian not having a gender-neutral pronoun, iCub was referred to with “him” in Italian (lui).

^7^ https://mathpickle.com/project/pollinator-puzzles/
