# Corrigendum: A Socially Adaptable Framework for Human-Robot Interaction

**DOI:** 10.3389/frobt.2021.812583

**Published:** 2021-12-14

**Authors:** Ana Tanevska, Francesco Rea, Giulio Sandini, Lola Cañamero, Alessandra Sciutti

**Affiliations:** ^1^ Department of Robotics, Brain and Cognitive Science, Italian Institute of Technology (IIT), Genova, Italy; ^2^ EECAiA Lab, School of Computer Science, University of Hertfordshire, Hatfield, United Kingdom; ^3^ Cognitive Architecture for Collaborative Technologies Unit, Italian Institute of Technology (IIT), Genova, Italy; ^4^ Neurocybernetics Team, ETIS Lab, CY Cergy Paris Université, ENSEA, CNRS UMR8051, Cergy-Pontoise, France

**Keywords:** human-robot interaction, social adaptability, affective interaction, personalized HRI, emotion recognition

In the published article, there was an error regarding the affiliations for Lola Cañamero. As well as having affiliation 2 “EECAiA Lab, School of Computer Science, University of Hertfordshire, Hatfield, United Kingdom”, they should also have 4 “Neurocybernetics Team, ETIS Lab, CY Cergy Paris Université, ENSEA, CNRS UMR8051, Cergy-Pontoise, France”.

The authors apologize for this error and state that this does not change the scientific conclusions of the article in any way. The original article has been updated.

